# Supplementary material for: Temperature-dependent dynamic disproportionation in LiNiO2
Source: Nat Commun. 2025 Oct 23;16:9379. doi: 10.1038/s41467-025-64429-4 (PMC12549827; doi:10.1038/s41467-025-64429-4)
Supplement: Supplementary file 1 — Supplementary Information [file 41467_2025_64429_MOESM1_ESM.pdf]

Temperature-Dependent Dynamic Disproportionation in $LiNiO_2$ Supplementary Information

Andrey D. Poletayev[1,2*], Robert J. Green[3,4], Jack E.N. Swallow[1,2], Lijin An[1,2], Leanne Jones[1,2], Grant Harris[3], Peter Bencok[5], Ronny Sutarto[6], Jonathon P. Cottom[2,7,8], Benjamin J. Morgan[2,7], Robert A. House[1,2], Robert S. Weatherup[1,2*], M. Saiful Islam[1,2,7*]

[1] Dept. of Materials, University of Oxford, Oxford, UK
[2] The Faraday Institution, Harwell Science and Innovation Campus, Didcot, UK
[3] Dept. of Physics and Engineering Physics, University of Saskatchewan, Saskatoon SK, Canada
[4] Stewart Blusson Quantum Matter Institute, Univ. of British Columbia, Vancouver BC, Canada
[5] Diamond Light Source, Harwell Science and Innovation Campus, UK
[6] Canadian Light Source, Saskatoon SK, Canada
[7] Dept. of Chemistry, University of Bath, Bath, UK
[8] Present: Advanced Research Center for Nanolithography, Amsterdam, The Netherlands
[*] correspondence to andrey.poletayev@gmail.com, robert.weatherup@materials.ox.ac.uk, saiful.islam@materials.ox.ac.uk

Calculated X-ray Absorption Spectra at the Ni $L_{3,2}$-edge

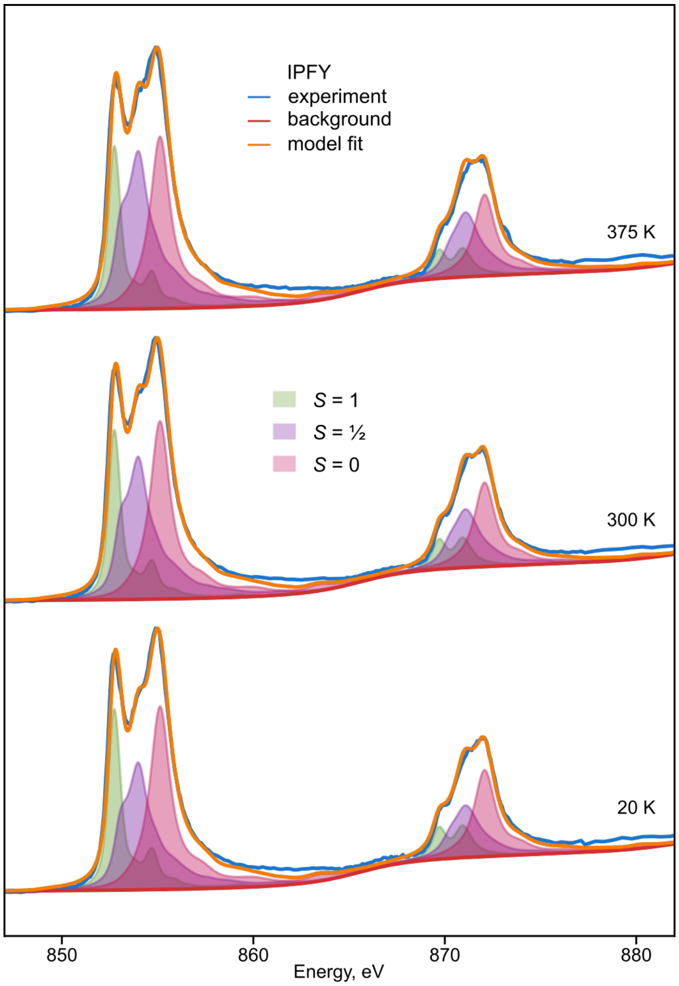

Supplementary Figure 1: Temperature Evolution of the IPFY spectra of $LiNiO_2$. The fitting of the IPFY spectra in Figure 2c with $S = ½$ fractions 34%, 38%, and 41% at 20 K, 300 K, and 375 K, respectively, and the excess of $S = 1$ species over the $S = 0$ species is 3% at all temperatures. The difference between the fits to these spectra, collected at CLS, and those in Figure 3, collected at DLS, is 2% in both parameters at 300 K. The increasing smoothness of the $LiNiO_2$ spectra at the energies where the spectrum of the $S = ½$ species has a maximum, in the middle of the edges, towards elevated temperature is further consistent with dynamic interconversion.

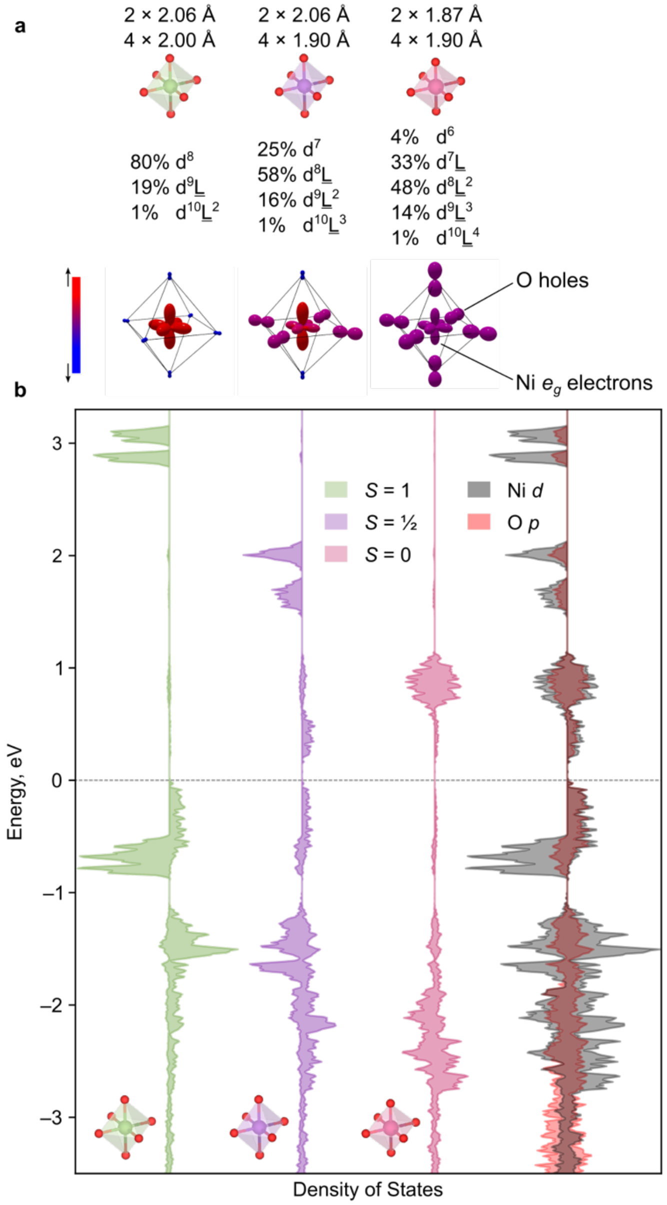

Supplementary Figure 2: Models of three Ni species in $LiNiO_2$. (a) Bond length and charge-transfer multiplet components for each species, and Ni e_g electron and ligand hole density matrix plots. Salient features are high ionicity and low ligand hole content for the ($S = 1$), preferred occupancy of the $3z^2–r^2$ orbitals and bonding anisotropy consistent with Jahn-Teller distortions ($S = ½$), and spin depolarization and ligand hole contributions ($S = 0$). (b) Projected densities of Ni 3d states for $S = 1$ (green), $S = ½$ (purple), and $S = 0$ (pink) Ni species near the Fermi level. Right: total Ni 3d (grey) and O 2p (red) densities of states. The spin depolarization of the $S = 0$ states near 1 eV is consistent with the density matrix from ligand field calculations.

Characterization and experimental controls

TEY, TFY, IPFY comparison of $LiNiO_2$ and $NaNiO_2$

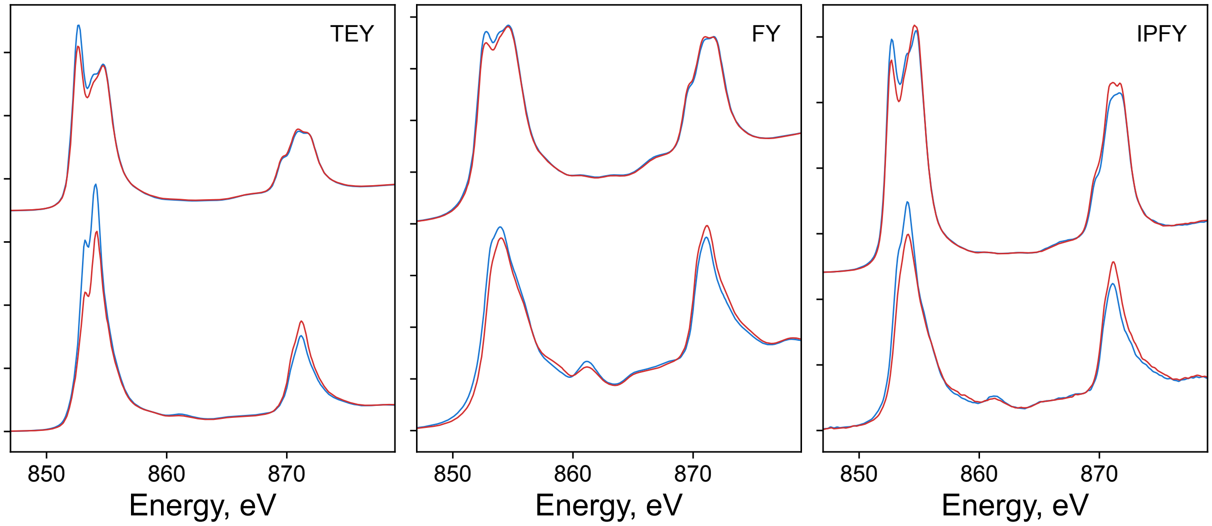

Supplementary Figure 3: Ni soft x-ray absorption spectroscopy using three detection modes. (a) total electron yield, (b) fluorescence yield, (c) inverse partial fluorescence yield via emission at the O K-edge. Blue: σ_r polarisation, red: σ_l polarisation. Top: $LiNiO_2$, 6 K, 8 T. Bottom: $NaNiO_2$, 10 K, 8 T.

Absence of XMLD at the Ni edge and absence of dichroism at the O K edge

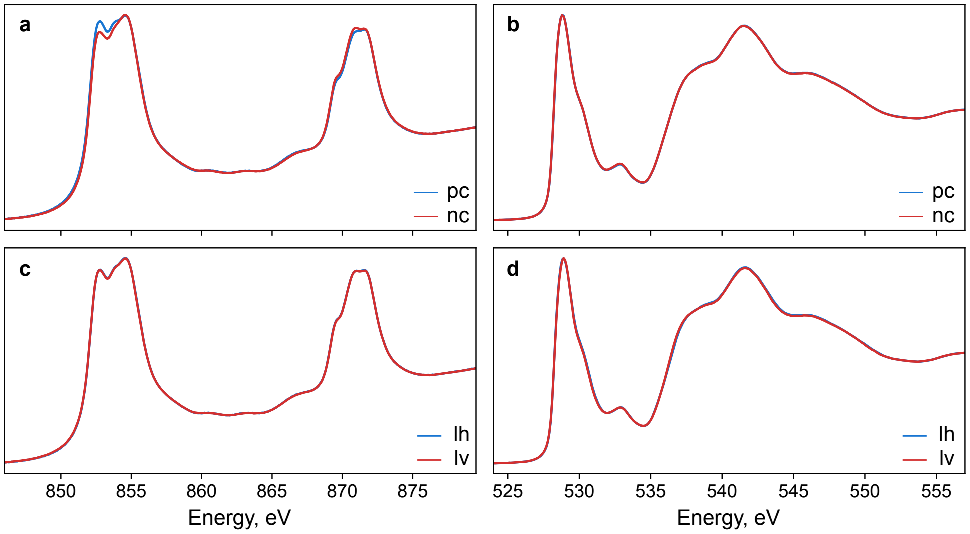

Supplementary Figure 4: x-ray circular and linear dichroism in $LiNiO_2$. (a) circular, Ni L-edge, (b) circular, O K-edge, (c) linear, Ni L-edge, (d) linear, O K-edge. All: fluorescence yield, 6 K, 8 T. Only circular dichroism is observable, and only at the Ni L-edge.

X-ray Diffraction of $LiNiO_2$ and $NaNiO_2$ samples

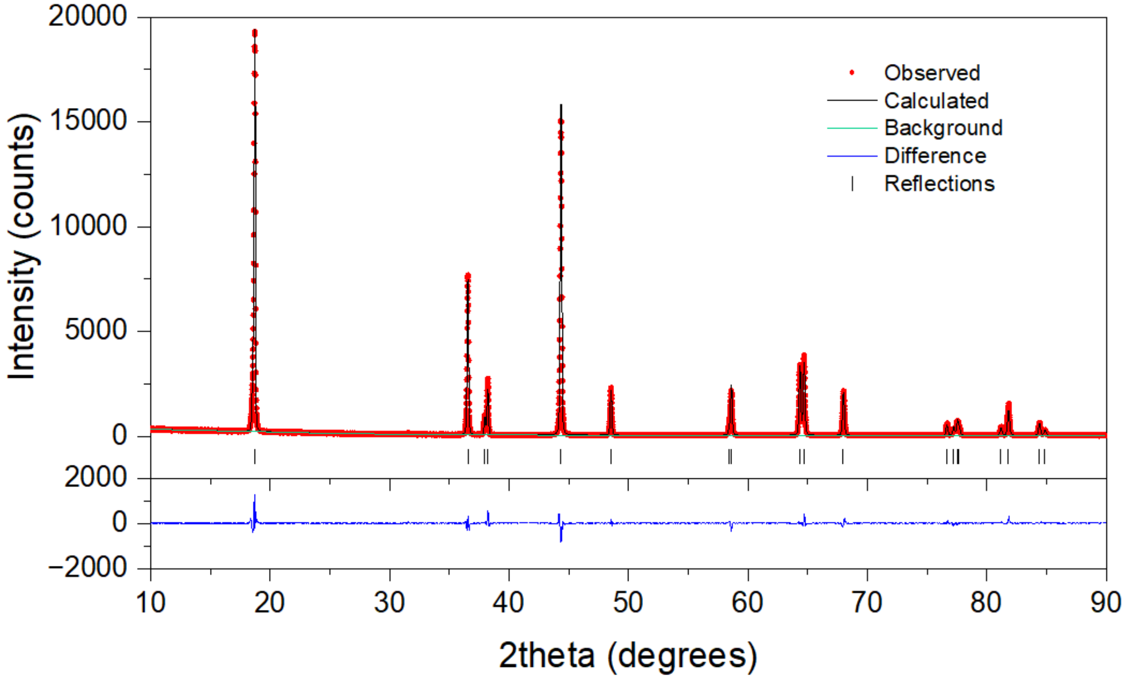

Supplementary Figure 5. X-ray diffraction data for $LiNiO_2$.

Supplementary Table 1. Rietveld refinement parameters for $LiNiO_2$.

	x	y	z	Occupancy	U_{iso}
Li	0.000	0.000	0.500	0.98(2)	0.006(2)
Ni	0.000	0.000	0.500	0.02(2)	0.013(2)
Ni	0.000	0.000	0.000	1.00	0.013(2)
O	0.000	0.000	0.260(1)	1.00	0.021(1)
Space Group: R-3m a = 2.879(1) c = 14.204(1)<br>G.O.F. = 1.38 R_w = 9.13					

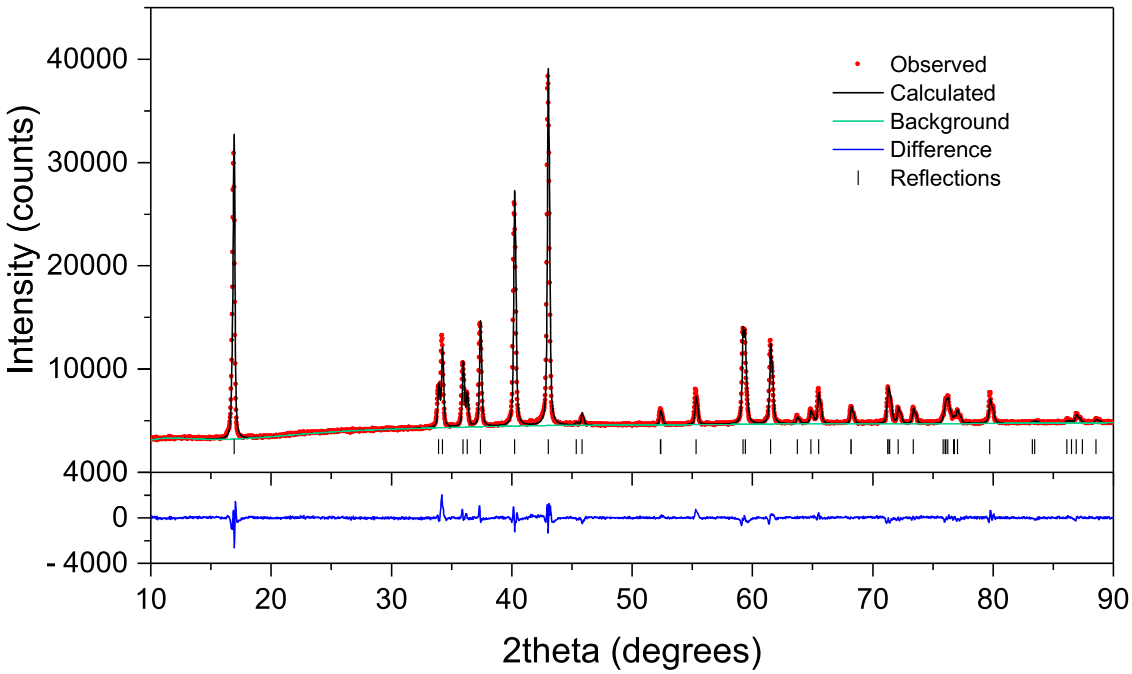

Supplementary Figure 6. X-ray diffraction data for $NaNiO_2$.

Supplementary Table 2. Rietveld refinement parameters for $NaNiO_2$.

	x	y	z	Occupancy	U_{iso}
Na	0.000	0.500	0.500	1.00	0.007(1)
Ni	0.000	0.000	0.000	1.00	0.031(1)
O	0.281(1)	0.000	0.791(1)	1.00	0.015(1)
Space Group: C2/m a = 5.327(1) b = 2.847(1) c = 5.587(1)<br>G.O.F. = 1.83 R_w = 2.59					

Calculated Resonant Inelastic X-ray Scattering Maps at the Ni $L_{3,2}$-edge

RIXS maps calculated from the charge-transfer multiplet model are shown in Supplementary Figure 7. The intensity near 857 eV photon energy and 2 eV loss is attributable to $S = 0$ Ni species.

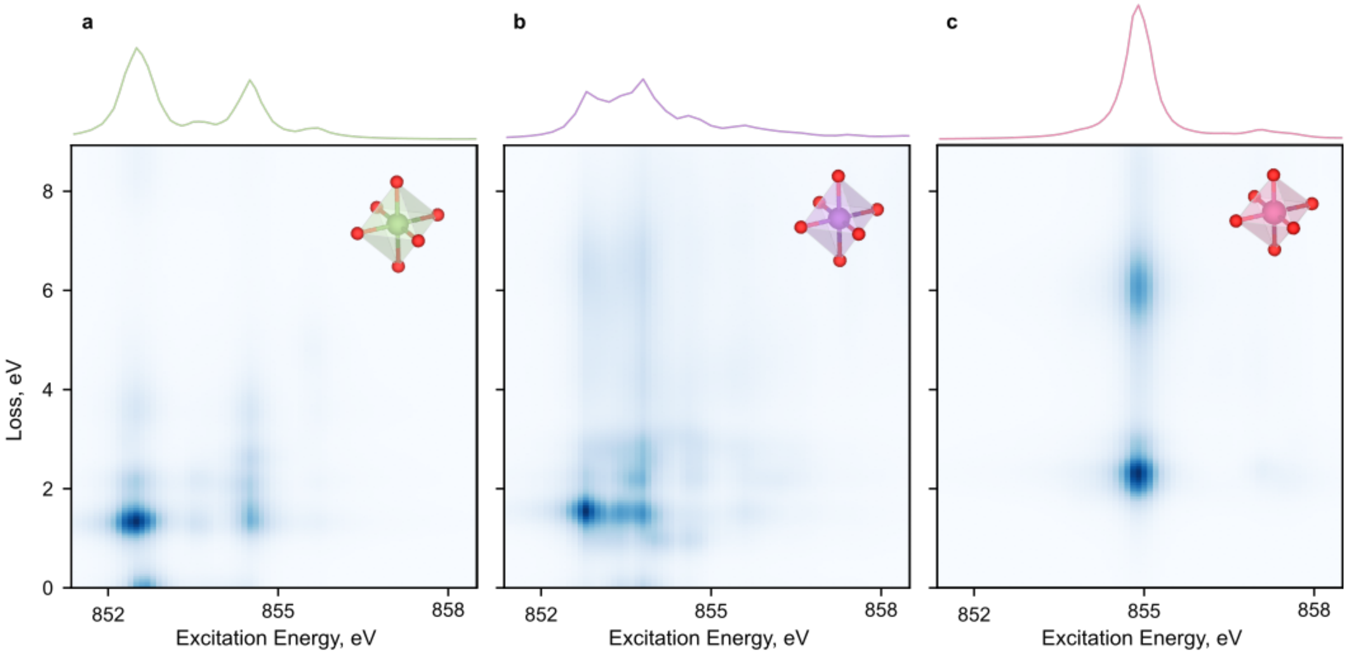

Supplementary Figure 7: Calculated localized transitions in the Ni L_3 edge RIXS of $LiNiO_2$. (a) $S = 1$ Ni species, (b) $S = ½$ Ni species, (c) $S = 0$ Ni species. The integrated spectra are plotted above the intensity maps for each panel.

Choice of density functional using thermodynamic data

The geometries of NiO and $LiNiO_2$ simulated in small cells are close to experimental values[1,2] and depend weakly on the choice of meta-GGA functionals (Supplementary Table 3). The predicted thermodynamics and the geometry of NiO_2 depend more significantly on the choice of functional. Less expensive approaches such as PBE+U were discarded for their inability to accurately represent the *c* lattice contraction of the delithiated NiO_2 phase[3]. Even at the meta-GGA level of theory, a dispersion correction is necessary to reproduce the *c* lattice contraction of NiO_2. Since fitted Hubbard *U* values are specific to oxidation states[4], empirical +*U* corrections were avoided. In principle, the unusually large low-temperature heat capacity of $LiNiO_2$ makes a larger than normal contribution to room-temperature energies at 0.15 eV/f.u. for heating from 20 K to 300 K[5], but this correction was not applied here. SCAN over-stabilizes the NiO and $LiNiO_2$ phases, whereas r^2SCAN offers a precise match to NiO thermodynamics, but both are used to perform ab initio molecular dynamics shown below. Overall, r^2SCAN+rVV10 best reproduced the thermodynamics of $LiNiO_2$ and its constituent phases[5,6] and the geometry of the delithiated NiO_2 without major outlier errors.

Disproportionation in geometry relaxations

The relaxed energy of the commonly computed zigzag $P2_1/c$ phase is 83 meV/f.u. below the bulk-average $R\overline{3}m$ phase, consistent with previous studies[8]. However, in relaxations starting with the

$R\overline{3}m$ phase we found a partial disproportionation of Ni spins from S = ½ to S = 1 and S = 0 for both SCAN and r^2SCAN meta-GGA functionals. If all S = ½ Ni species are consumed and the resulting (S = 1, S = 0) pairs ordered linearly, such disproportionation yields the P2/c phase, previously predicted computationally[8–10]. The similarity between the three interpenetrating transition-metal sublattices in the ground state of Li(NiMnCo)O_2[11], the ground state of noncollinear spin models[12], and the disproportionated structure of $AgNiO_2$[13,14], which each exhibit three interpenetrating sublattices within the hexagonal layer of TMO_6 octahedra, suggests that the logical limit for disproportionation could be a phase where Ni species with spins S = 1, S = ½, and S = 0 similarly occupy three sublattices, a static version of which was proposed by Foyevtsova *et al.*[10].

When this asymptotic three-fold disproportionated structure is relaxed, the volumes of the NiO_6 octahedra correlate with the spins of the Ni species: 10.9 Å^3 for S = 1, 9.9 Å^3 for S = ½, and 9.0 Å^3 for S = 0 (Figure 2a), similar to $AgNiO_2$. All octahedra are somewhat distorted. The longest relaxed Ni–O bond is 2.06 Å, which is shorter than the Ni–O distance of 2.10 Å relaxed in the $P2_1/c$ structure, and consistent with structural probes[15]. The relaxed limiting three-fold disproportionated structure and the two-fold disproportionated P2/c structure have energies 14 meV/f.u. and 12 meV/f.u. above the zigzag $P2_1/c$ structure, respectively. The energetic penalty for changing the ordering (clockwise vs anti-clockwise) of the three sublattices within adjacent NiO_2 layers is 5 meV/f.u., and antiferromagnetic (AFM) or ferrimagnetic (FiM) orderings carry penalties of about 1 meV/f.u. Bader charges in threefold disproportionated $LiNiO_2$ are 1.40, 1.53, and 1.66 for Ni, and vary between 1.24-1.29 for O. In O3-stacked NiO_2, the Bader charges are 1.68 and 0.84 for Ni and O, respectively.

Configurational entropy is expected to favor the three-fold disproportionated structure and other intermediate compositions over the fully comproportionated ($P2_1/c$ or $NaNiO_2$-like monoclinic C2/m) and two-fold disproportionated P2/c endpoints. The cluster expansion model used in Figure 2d identifies the comproportionated all-spin-half $LiNiO_2$ as 11 meV/f.u. lower in energy than the three-fold disproportionated structure but does not predict it to be observable. The full exploitation of the cluster expansion model is the subject of follow-on work.

Supplementary Table 3: Comparison between structural and thermodynamic parameters of $LiNiO_2$ simulated with meta-GGA functionals and their experimental values.

Parameter	SCAN	SCAN +rVV10	r^2SCAN	r^2SCAN +rVV10	Exp't	Ref.
$R\overline{3}m\, a$, Å	2.85	2.84	2.85	2.85	2.878	1
$R\overline{3}m\, c$, Å	14.10	14.05	14.10	14.04	14.19	1
$R\overline{3}m\, \Delta H$, eV	-6.62	-6.74	-6.23	-6.40	-6.15	6
NiO a, Å	4.155	4.146	4.16	4.15	4.17	2
NiO ΔH, eV	-2.73	-2.80	-2.41	-2.51	-2.48	6
Li_2O ΔH, eV	-6.16	-6.21	-6.04	-6.09	-6.20	6
NiO_2 a, Å	2.77	2.77	2.78	2.78	2.81	7
NiO_2 c, Å	13.67	13.08	13.63	13.02	13.3	7

Dependence of Ni spin disproportionation on the choice of density functional

In the AIMD simulations of bulk pristine $LiNiO_2$, or bulk $LiNiO_2$ with an antisite Ni_{Li} defect, substantial variation in spin was observed only for Ni. Spins on oxygens never exceeded ≈ 0.145 μ_B. However, the temperature dependence of the free energy as projected onto the Ni spin coordinate differed substantially with the choice of k-points and meta-GGA functionals.

First, we compare the free-energy surfaces from the reference simulation performed with a 2×2×2 k-point mesh (Supplementary Figure 8a, blue) versus a simulation performed at the Γ point exclusively (Supplementary Figure 8a, pink) with the r^2SCAN functional. The two free-energy surfaces are drastically different: the Γ-point simulation does not predict stable disproportionation or any thermally activated dynamics. In the Γ-point simulation, magnetic moments below 0.2 μ_B or above 1.5 μ_B are not realized. Supplementary Figure 8a also shows that a small distortion of the cell angles acquired during the relaxation of the three-spin disproportionated structure perturbs the energy surface (Supplementary Figure 8a, purple), making transitions slightly rarer relative to the symmetric hexagonal high-temperature cell (Supplementary Figure 8a, blue). Performing the simulation in the isobaric ensemble has a similar effect (Supplementary Figure 8a, green), and using a larger cell with 144 atoms instead of 108 as in all the other runs (Supplementary Figure 8a, orange) predicts slightly smaller activation energies. Overall, the inclusion of off-zone-center k-points is critical to reproducing experimental observables.

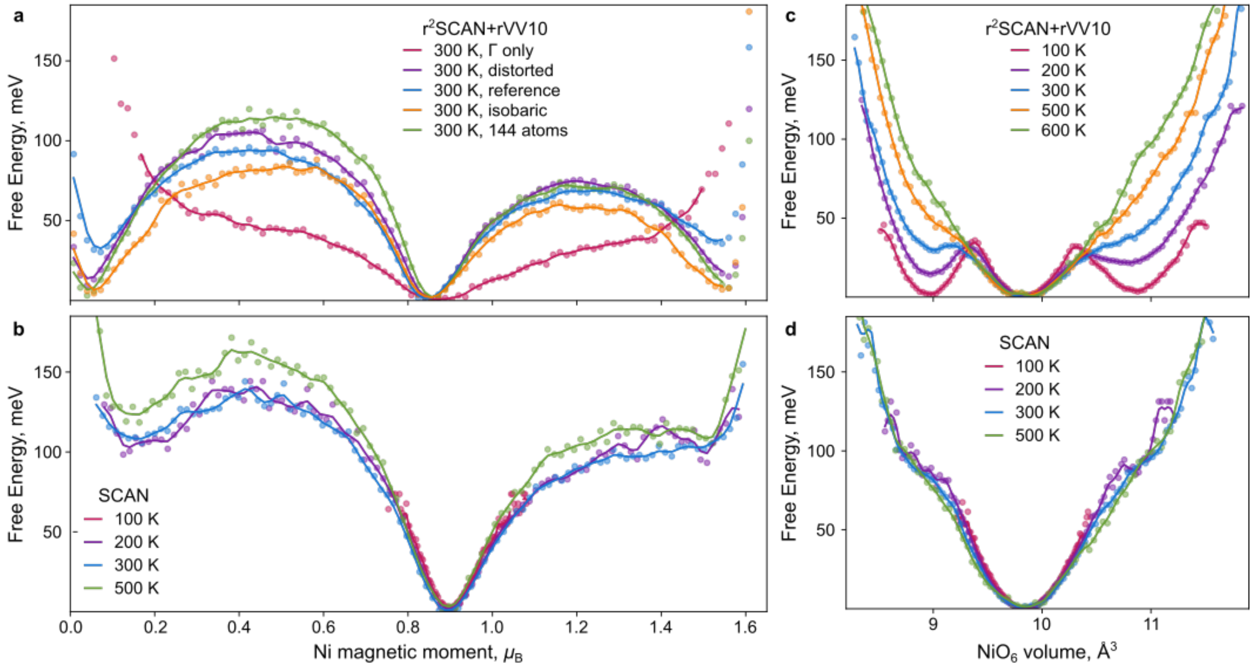

Supplementary Figure 8: Sensitivity of simulated free energy surfaces to simulation parameters. (a) free energy surfaces versus the Ni magnetic moment with r^2SCAN+rVV10 at 300 K: reference as in Figure 3 (blue), relaxed cell with a small distortion (purple), hexagonal cell at the Γ point only (pink), hexagonal cell with 144 atoms (green), and simulation in the isobaric ensemble (orange). (b) free energy surfaces versus the Ni magnetic moment with SCAN and the hexagonal cell. (c-d) free energy surfaces versus the NiO_6 volume with r^2SCAN+rVV10 (c) or SCAN (d) and the hexagonal cell.

Using the SCAN functional (Supplementary Figure 8b) yields nearly temperature-independent free-energy surfaces, in contrast with r^2SCAN. SCAN favors all-spin-half arrangements upon cooling, while r^2SCAN favors disproportionation at cryogenic temperatures. The results from all functionals (r^2SCAN and SCAN+rVV10 not shown) agree best at high temperatures and disagree most towards cryogenic temperatures. This can be further seen in the projections of free energy surfaces onto the NiO_6 octahedral volumes in Supplementary Figure 8c for r^2SCAN+rVV10 and Supplementary Figure 8d for SCAN. This behavior of the simulated dynamics follows the trends in matching the thermodynamics of NiO and $LiNiO_2$ (Supplementary Table 3): the choice of functional dominates the thermodynamic ensemble, simulation cell size, and non-local dispersion corrections.

We hypothesize that the sensitivity to the choice of functional is due to the small relative energy differences under investigation (<20 meV/f.u. between all relevant relaxed structures) being dominated by phonon energies (>60 meV) and the differences in predicted thermodynamic formation energies across functionals (e.g., 0.3–0.4 eV/f.u. for NiO, Supplementary Table 3). This may represent a limit of precision for computational studies of $LiNiO_2$ structure and dynamics.

Structural Descriptors

The key observable structural signatures of local geometry are the unit cell parameters (Supplementary Table 3) and the Ni-O bond length, which are typically probed by EXAFS[16–18] and diffraction[15]. These methods probe the average structure, and in both cases a fitting or refinement is required to obtain structural information. Several aspects complicate the use of structural information for fingerprinting Ni species: the primacy of the electronic coordinate, the noise in the structural coordinate, and the necessary regularization in interpreting experimental data. The electronic coordinate provides a stronger and lower-noise separation of the three Ni species (main text and Supplementary Figure 8), and the NiO_6 octahedra of multiple species have the same bond lengths in static calculations. Despite drastically different predictions of Ni electronic speciation (Supplementary Figure 8ab), the SCAN and r^2SCAN functionals yield similar unit cell geometries (Supplementary Table 3) and distributions of Ni-O bond lengths (Supplementary Figure 9). The low-temperature bond length distributions are sharpest and provide the strongest contrast between functionals. The r^2SCAN structures appear stiffer with narrower bond length distributions and have

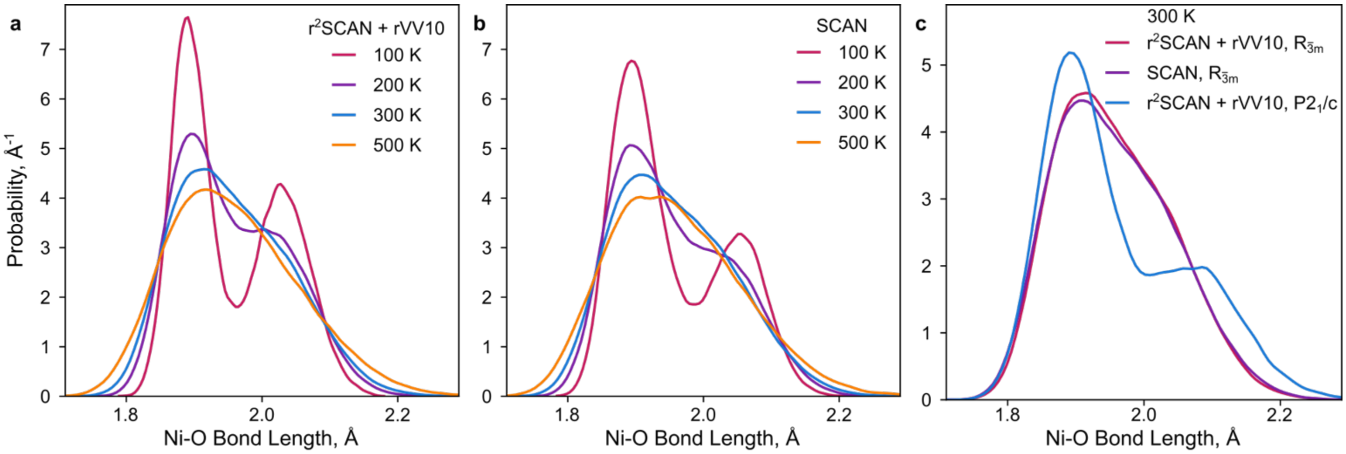

Supplementary Figure 9: Simulated Ni-O bond distances in $LiNiO_2$. (a) simulated with r2SCAN+rVV10 as a function of temperature, (b) simulated with SCAN as a function of temperature (c) at 300 K for two unit cells.

shorter long bonds. However, between 200 K and 300 K the two distinct bond populations merge so that by 300 K, the distributions of bond lengths are virtually indistinguishable if the simulations are carried out in cell geometries relaxed from the hexagonal unit cell (Supplementary Figure 9c), despite differences in the predicted electronic structure. Both hexagonal simulations yield long bonds at ≈2.05 Å, in close agreement with experiment, for both electronic structures. By contrast, the zigzag structure ($P2_1/c$ in Supplementary Figure 9c) predicts long bonds at ≈2.10Å, which is longer than any experimentally obtained ones.

Spin Transition Statistics

To verify whether the AIMD trajectories were of sufficient length to construct free energy surfaces, transitions between spin states were counted for each Ni ion by milestoning at 200 K and above (Methods). Simulations were continued until every ion had exhibited multiple transitions (Supplementary Figure 10). Excluding the initial 1 ps for thermostat equilibration, the simulations at 200 K and 300 K were collected for over 10 ps with the longest residence times 7.7 ps at 200 K and 2.7 ps at 300 K. The trajectories at 500 K and 600 K were collected for at least 4.6 ps with the longest residence times 1.4 ps. At 300 K, the fastest transition times are on the order of 50 fs, close to one period of the Raman-active vibrations of Ni–O bonds[19]. This is consistent with the coupling between NiO_6 geometry and Ni states: geometry changes mediated by vibrations set a speed limit on changes in electronic states.

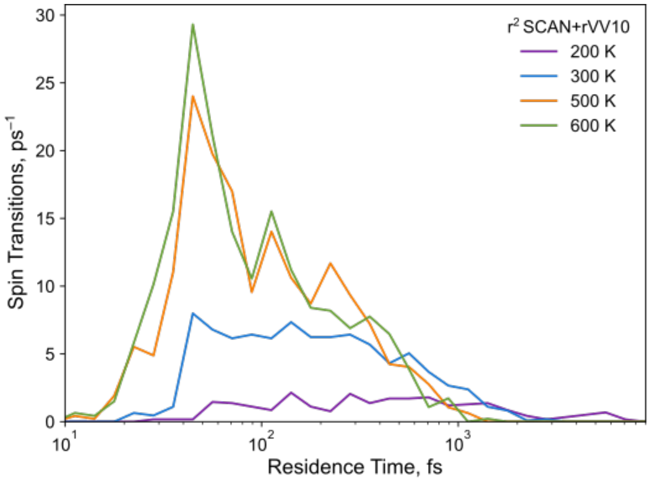

Supplementary Figure 10: Spin transitions in ab initio molecular dynamics. The frequency of spin transitions is plotted versus the length of time the Ni ions spend in a particular spin state before making a transition.

Defect chemistry and stabilization of the Ni_{Li} antisite

We next explore the influence of disproportionation on defect chemistry via the localization of electronic charge carriers and the intrinsic antisite defect. Hole and electron polarons are relaxed in the limiting three-fold disproportionated structure by distorting the S = ½ Ni octahedra to sizes commensurate with S = 0 and S = 1, respectively. While distortions of neighboring octahedra are minimal, some relaxations instead yield a reordering of the Ni spins and their respective octahedral volumes. Results are consistent between 108-atom and 144-atom supercells. The stabilization of polarons is consistent with a correspondence between the spin states and formal charge states of Ni species. By contrast, we have not been able to stabilize a hole polaron or a Ni with $S \approx 0$ in the $P2_1/c$ structure at the meta-GGA level of theory, except in a 2×3×2 supercell (96 atoms) and surrounded by six opposite-sign S = -½ spins on adjacent Ni ions, which forms a partial superlattice like the three-fold disproportionation discussed here. This arrangement is clearly artificial, is energetically unfavorable relative to a delocalized hole, and is not stabilized in other supercells. Electron polarons are stable as S = 1 Ni in the $P2_1/c$ structure, consistent with earlier work[20].

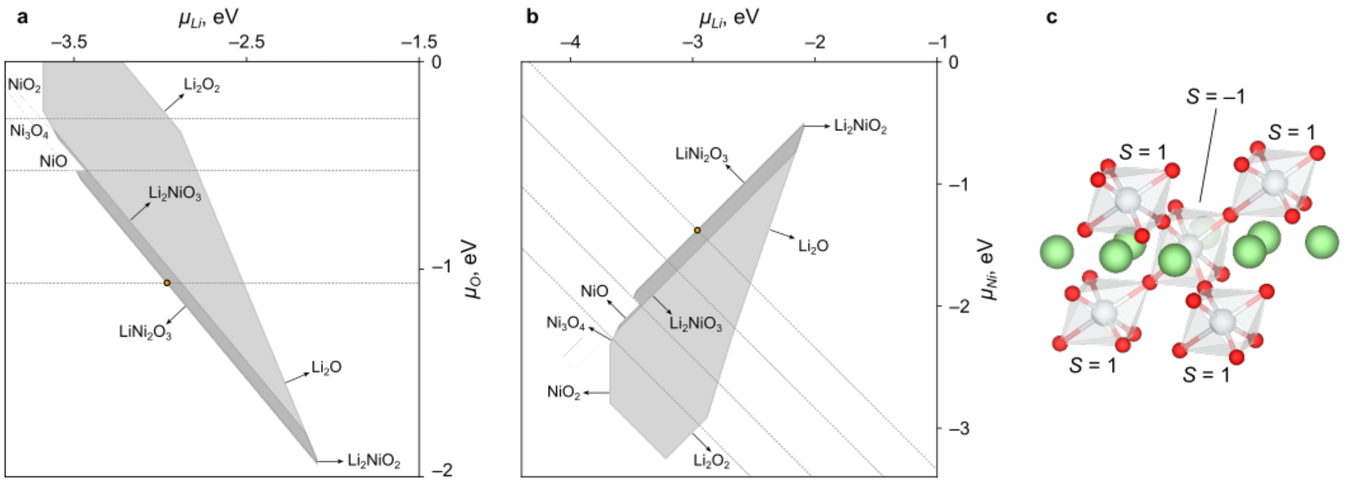

Supplementary Figure 11: Phase and defect stability in $LiNiO_2$. (a,b) thermodynamic phase stability of disproportionated $LiNiO_2$, with competing phases highlighted, and the synthetic conditions denoted by an orange circle. (c) Antiferromagnetic stabilization of the Ni_{Li} antisite defect by S = 1 Ni ions.

To calculate the formation energies for the Ni_{Li} antisite defect, we first determine the chemical potentials of the elements from reference phase energies[21] (Supplementary Figure 11ab). Accounting for the paramagnetic transition of NiO and representing the energetics of NiO accurately (Supplementary Table 3) are key to estimating μ_{Li} and μ_{Ni} and the defect formation energy. The phase equilibrium at synthesis is between $LiNiO_2$ and a disordered rocksalt, computationally predicted as $LiNi_2O_3$[22]. This is consistent with the experimental synthesis pathway from NiO via lithium insertion followed by the formation of ordered layers[23]. Finally, since layered Li_2NiO_3 is not easily synthesized[24], and not observed as a decomposition product, we include an additional region of likely metastability over Li_2NiO_3 (light grey in Supplementary Figure 11ab).

Within disproportionated structures, the energy of the Ni_{Li} antisite depends on the arrangements of the nickel spins adjacent to it within the NiO_2 layers. The lowest-energy configuration is one where S = –1 Ni_{Li} is surrounded by S = 1 Ni ions via 180-degree Ni–O–Ni motifs (Supplementary Figure 11c), reminiscent of antiferromagnetic NiO. At synthesis conditions, the formation energies of multiple configurations are negative, with the lowest at –60 meV, whereas the lowest defect energy in the $P2_1/c$ structure is 220 meV. In the $P2_1/c$ structure, only one Ni_{Ni} carries S = 1 to compensate the introduction of the Ni_{Li}. Our estimate of the formation energies for the most stable local configurations of Ni_{Li} is lower than the already low figures reported earlier[20] due to accounting for this additional antiferromagnetic stabilization enabled by disproportionation in $LiNiO_2$.

While the local atomic and spin arrangements are expected to fluctuate rapidly at synthesis, the results remain relevant for room-temperature degradation pathways during battery operation. In AIMD trajectories incorporating a Ni_{Li} antisite, the Ni ions sharing octahedral corners with the defect as in Supplementary Figure 11c carry S = 1 on average 50% more often than Ni ions away from the defect: the S = 1 that compensate the antisite are not free even at elevated temperatures. We conclude that magnetic stabilization contributes to the ubiquity of this pervasive defect. Ni_{Li} is stable not only because of the size match between Ni and Li, but also because of the ability of the Ni to disproportionate in the layered structure, which distinguishes it from Co and Mn oxides, and

from the monoclinic $NaNiO_2$. This effect likely contributes to the driving forces for surface reconstructions and Ni migration into the Li layer during charging.

References

1. Dyer, L. D., Borie, B. S. & Smith, G. P. Alkali Metal-Nickel Oxides of the Type $MNiO_2$. *J Am Chem Soc* **76**, 1499–1503 (1954).
2. Cheetham, A. K. & Hope, D. A. O. Magnetic ordering and exchange effects in the antiferromagnetic solid solutions $Mn_xNi_{1-x}O$. *Phys Rev B* **27**, 6964–6967 (1983).
3. Chakraborty, A., Dixit, M., Aurbach, D. & Major, D. T. Predicting accurate cathode properties of layered oxide materials using the SCAN meta-GGA density functional. *NPJ Comput Mater* **4**, 46–49 (2018).
4. Kowalski, P. M. *et al.* Fundamentals of energy storage from first principles simulations: Challenges and opportunities. *Front Energy Res* **10**, 1–14 (2023).
5. Kawaji, H. *et al.* Low-temperature heat capacity of layer structure lithium nickel oxide. *SSI* **152**, 195 (2002).
6. Wang, M. & Navrotsky, A. Enthalpy of formation of $LiNiO_2$, $LiCoO_2$ and their solid solution, $LiNi_{1-x}Co_xO_2$. *Solid State Ion* **166**, 167–173 (2004).
7. Mock, M., Bianchini, M., Fauth, F., Albe, K. & Sicolo, S. Atomistic understanding of the $LiNiO_2$–NiO_2 phase diagram from experimentally guided lattice models. *J Mater Chem A* **9**, 14928–14940 (2021).
8. Sicolo, S., Mock, M., Bianchini, M. & Albe, K. And Yet It Moves: $LiNiO_2$, a Dynamic Jahn–Teller System. *Chemistry of Materials* **32**, 10096–10103 (2020).
9. Chen, H., Freeman, C. L. & Harding, J. H. Charge disproportionation and Jahn-Teller distortion in $LiNiO_2$ and $NaNiO_2$: A density functional theory study. *Phys Rev B* **84**, 1–7 (2011).
10. Foyevtsova, K., Elfimov, I., Rottler, J. & Sawatzky, G. A. $LiNiO_2$ as a high-entropy charge- and bond-disproportionated glass. *Phys Rev B* **100**, 165104 (2019).
11. Kunnikuruvan, S., Chakraborty, A. & Major, D. T. Monte Carlo- and Simulated-Annealing-Based Funneled Approach for the Prediction of Cation Ordering in Mixed Transition-Metal Oxide Materials. *The Journal of Physical Chemistry C* **124**, 27366–27377 (2020).
12. Miyashita, S. A Variational Study of the Ground State of Frustrated Quantum Spin Models. *J Physical Soc Japan* **53**, 44–47 (1984).
13. Wawrzyńska, E. *et al.* Charge disproportionation and collinear magnetic order in the frustrated triangular antiferromagnet $AgNiO_2$. *Phys Rev B* **77**, (2008).
14. Pascut, G. L. *et al.* Direct observation of charge order in triangular metallic $AgNiO_2$ by single-crystal resonant x-ray scattering. *Phys Rev Lett* **106**, 2–5 (2011).
15. Chung, J.-H. *et al.* Local structure of $LiNiO_2$ studied by neutron diffraction. *Phys Rev B* **71**, 064410 (2005).
16. Rougier, A., Delmas, C. & Chadwick, A. V. Non-cooperative Jahn-Teller effect in $LiNiO_2$: An EXAFS study. *Solid State Commun* **94**, 123–127 (1995).
17. Nakai, I., Takahashi, K., Shiraishi, Y., Nakagome, T. & Nishikawa, F. Study of the Jahn–Teller Distortion in $LiNiO_2$, a Cathode Material in a Rechargeable Lithium Battery, by in Situ X-Ray Absorption Fine Structure Analysis. *J Solid State Chem* **140**, 145–148 (1998).
18. Huang, H. *et al.* Unusual double ligand holes as catalytic active sites in $LiNiO_2$. *Nat Commun* **14**, 2112 (2023).
19. Flores Cedeño, E. J. Development of operando diagnostics for Li-ion cathodes by Raman spectroscopy. (ETH Zürich, 2019). doi:10.3929/ethz-b-000373382.
20. Hoang, K. & Johannes, M. D. Defect chemistry in layered transition-metal oxides from screened hybrid density functional calculations. *J Mater Chem A* **2**, 5224–5235 (2014).
21. Buckeridge, J., Scanlon, D. O., Walsh, A. & Catlow, C. R. A. Automated procedure to determine the thermodynamic stability of a material and the range of chemical potentials necessary for its formation relative to competing phases and compounds. *Comput Phys Commun* **185**, 330–338 (2014).
22. Das, H., Urban, A., Huang, W. & Ceder, G. First-Principles Simulation of the (Li-Ni-V)O Phase Diagram and Its Relevance for the Surface Phases in Ni-Rich Li-Ion Cathode Materials. *Chem. Mater.* **29**, 7840–7851 (2017).
23. de Biasi, L. *et al.* Phase Transformation Behavior and Stability of $LiNiO_2$ Cathode Material for Li-Ion Batteries Obtained from In Situ Gas Analysis and Operando X-Ray Diffraction. *ChemSusChem* **12**, 2240–2250 (2019).
24. Bianchini, M. *et al.* From $LiNiO_2$ to Li_2NiO_3: Synthesis, Structures and Electrochemical Mechanisms in Li-Rich Nickel Oxides. *Chemistry of Materials* **32**, 9211–9227 (2020).
